# In Reply: Barriers to Radiomics Adoption for Urological Cancer Diagnosis in Low-Income and Middle-Income Countries: A Perspective from Pakistan

**DOI:** 10.1016/j.mcpdig.2025.100263

**Published:** 2025-09-09

**Authors:** Isaiah Z. Yao, Min Dong, William Y.K. Hwang

**Affiliations:** Hwa Chong Institution, Junior College Section, Singapore; Department of Haematology, National Cancer Centre Singapore, SingHealth, Singapore; Department of Haematology, Singapore General Hospital, SingHealth, Singapore

*To the Editor:*

We thank Ayub et al^1^ for their thoughtful letter in response to our recent article, “Deep Learning Applications in Clinical Cancer Detection: A Review of Implementation Challenges and Solutions”. Their contribution provides a valuable perspective from Pakistan and highlights the realities of implementing radiomics in low-resource healthcare systems, especially for urological cancers.^2^

In our review, we emphasized that barriers such as lack of local validation, infrastructural limitations, and insufficient training pipelines significantly impede adoption of artificial intelligence and radiomics across diverse settings. Ayub et al^1^ observations reinforce these points with compelling examples from clinical practice, emphasizing the urgent need for context-specific validation, particularly where imaging modalities and health care infrastructure differ substantially from high-income countries.

We especially appreciate their highlighting of 1.5T magnetic resonance imaging and limited imaging sequence availability, which echoes our discussion on technological mismatches between training environments and real-world deployment. Their emphasis on constrained funding priorities also resonates with our review, where we stressed that radiomics is often deprioritized in health systems managing competing infectious and chronic disease burdens.

Importantly, their letter advances the discussion by calling for open-source, low-cost radiomics workflows and cloud-based platforms to address these disparities. This is very much aligned with the solutions we proposed in our article, where we argued for international collaboration, equitable dataset sharing, and investment in local capacity building. We agree that without such collective efforts, the digital divide in cancer care will continue to widen.

In conclusion, we thank Ayub et al^1^ for their insightful correspondence, which both validates and expands on the themes of our review. Their letter represents an important contribution from a low-income and middle-income country perspective. By highlighting on-the-ground realities and practical pathways forward, their commentary strengthens the ongoing dialogue on ensuring that radiomics fulfills its global promise in cancer care.

## Potential Competing Interests

The authors report no competing interests.
